# The Central Role of the Ubiquitin–Proteasome System in EBV-Mediated Oncogenesis

**DOI:** 10.3390/cancers14030611

**Published:** 2022-01-26

**Authors:** Yonggang Pei, Erle S. Robertson

**Affiliations:** 1 School of Public Health and Emergency Management, Southern University of Science and Technology, Shenzhen 518055, China; peiyg@sustech.edu.cn; 2 Departments of Otorhinolaryngology-Head and Neck Surgery, and Microbiology, and the Abramson Cancer Center, Perelman School of Medicine at the University of Pennsylvania, Philadelphia, PA 19104, USA

**Keywords:** ubiquitin–proteasome system, EBV, oncogenesis

## Abstract

**Simple Summary:**

Epstein–Barr virus (EBV) is the first discovered human tumor virus, which contributes to the oncogenesis of many human cancers. The ubiquitin–proteasome system is a key player during EBV-mediated oncogenesis and has been developed as a crucial therapeutic target for treatment. In this review, we briefly describe how EBV antigens can modulate the ubiquitin–proteasome system for targeted protein degradation and how they are regulated in the EBV life cycle to mediate oncogenesis. Additionally, the developed proteasome inhibitors are discussed for the treatment of EBV-associated cancers.

**Abstract:**

Deregulation of the ubiquitin–proteasome system (UPS) plays a critical role in the development of numerous human cancers. Epstein–Barr virus (EBV), the first known human tumor virus, has evolved distinct molecular mechanisms to manipulate the ubiquitin–proteasome system, facilitate its successful infection, and drive opportunistic cancers. The interactions of EBV antigens with the ubiquitin–proteasome system can lead to oncogenesis through the targeting of cellular factors involved in proliferation. Recent studies highlight the central role of the ubiquitin–proteasome system in EBV infection. This review will summarize the versatile strategies in EBV-mediated oncogenesis that contribute to the development of specific therapeutic approaches to treat EBV-associated malignancies.

## 1. Introduction

Ubiquitin is a 76-amino acids polypeptide that is highly conserved in eukaryotic cells. Ubiquitination is a type of post-translational modification that targets specific proteins by covalent ligation to ubiquitin. Ubiquitination is tightly mediated by three families of ubiquitin-specific proteases: these are the ubiquitin-activating enzyme (E1), the ubiquitin-conjugating enzyme (E2), and the ubiquitin-protein ligase (E3). Briefly, ubiquitin is activated by the activating E1 in an ATP-dependent manner to form a thioester bond between ubiquitin covalently bound to E1. Secondly, the conjugating E2 transfers the activated ubiquitin from E1 to form an intermediate molecule. Thirdly, the E3 ligase then catalyzes the covalent bond of ubiquitin to the target substrate. This multi-step process is critical for modulation of diverse biological processes, including cell cycle, cell apoptosis, transcriptional regulation, and signal transduction [1]. The modification of substrates by a single ubiquitin is called monoubiquitination and is mostly associated with signal transduction, while the modification of the targeted protein by a ubiquitin chain is referred to as polyubiquitination, which can be recognized by the 26S proteasome for proteasomal degradation [2]. In the process of polyubiquitination, seven lysines located in the ubiquitin polypeptide can be utilized to form polyubiquitin chains that lead to various functions. Specifically, the well-studied K48 and K63-linked polyubiquitin chains are often involved in protein degradation and signal transduction, respectively [2,3]. Ubiquitination can also be reversed by deubiquitinating enzymes (DUBs), and it is important to note that the dysregulation of DUBs is highly linked to many human diseases [1,4].

Epstein–Barr virus (EBV) is the first discovered human oncogenic virus and infects more than 90% of the human population worldwide. It is closely associated with a broad spectrum of human malignancies, including Burkitt’s lymphoma (BL), Hodgkin lymphoma (HL), nasopharyngeal carcinoma (NPC), and gastric carcinoma (GC) [5,6]. These diseases are tightly linked to EBV lytic or latent infection, in which multiple viral antigens are specifically expressed. These transcription programs hijack different cellular host factors with various mechanisms to induce oncogenesis. In particular, EBV nuclear antigens (EBNA1, 2, 3A, 3B, 3C, leader protein), and latent membrane proteins (LMP1, 2A, 2B) have been shown to interact with the ubiquitin–proteasome system to manipulate cellular processes indispensable for EBV-mediated oncogenesis [7,8]. In this review, we highlight the strategies used by EBV antigens to manipulate the ubiquitin–proteasome pathway to target cellular host factors. Similarly, viral antigens can be modulated by ubiquitination in EBV-induced oncogenesis. These proteins may also serve as specific targets to facilitate the development of novel therapeutic strategies for targeted interventions against EBV-associated cancers.

## 2. EBV Latent Antigens Manipulate the Ubiquitin–Proteasome System for Targeted Protein Degradation

**Figure 1:**
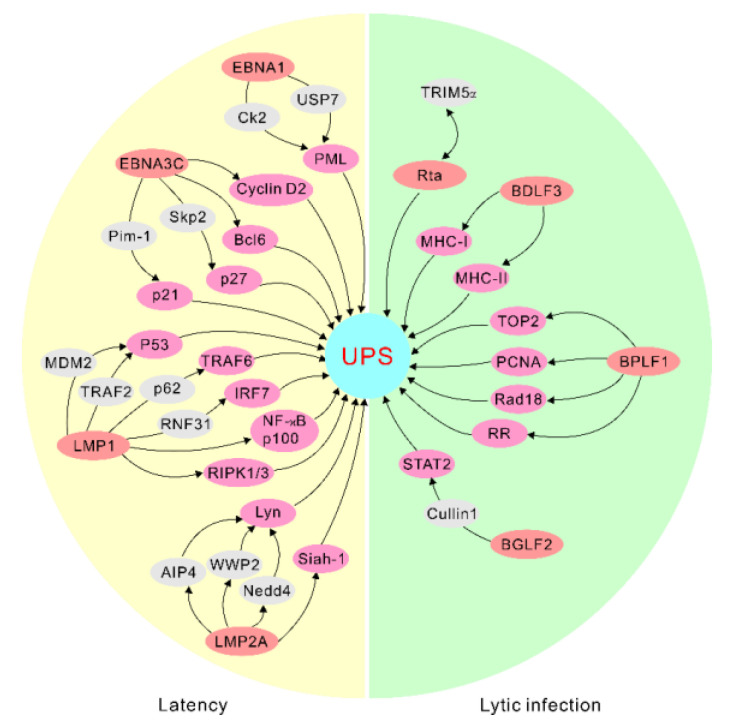
EBV antigens manipulate the ubiquitin–proteasome system for targeted protein degradation during latency and lytic infection. The representative interactions are highlighted. UPS, ubiquitin–proteasome system.

EBV latent programs are characterized by the expression of viral latent antigens. These EBV-encoded proteins can mediate the degradation of cellular factors through the ubiquitin–proteasome system to induce oncogenesis in EBV-infected cells (Table 1). A previous chemistry-based functional proteomic screen identified active ubiquitin-specific proteases (USP) in EBV-infected cells [9]. USP5/IsoT, USP7/HAUSP, USP9, and USP15i are higher expressed in EBV-transformed lymphoblastoid cell line (LCL) than in the Burkitt’s lymphoma cell line Raji [9]. This suggests several EBV latent antigens (e.g., EBNA2, EBNA3s, and LMPs) expressing in LCL cells but not Raji cells may induce expression of these UPSs that play potential roles in EBV-mediated lymphomagenesis. Using affinity chromatography in vitro and tandem affinity purification (TAP)-tagging in vivo approaches, the EBV nuclear antigen 1 (EBNA1) was identified to be associated with several cellular proteins, such as USP7/HAUSP, CK2, PRMT5 [10]. Further studies showed that EBNA1 is associated with host USP7 for PML disruption [11] (Figure 1). EBNA1 also recruits the cellular CK2 kinase to directly interact with PML proteins and promotes CK2-mediated PML phosphorylation, which induces the polyubiquitylation and degradation of PML [11]. Additionally, both EBNA1 and p53 can bind to the same domain of USP7, and the competitive binding of EBNA1 to USP7 reduces p53 stability and facilitates cell survival in EBV-infected cells [12,13].

The latent EBV nuclear antigen 3C (EBNA3C) is essential for transformation of human primary B lymphocytes in vitro [14]. EBNA3C manipulates several cellular proteins through their targeted degradation by the ubiquitin–proteasome system to facilitate cell proliferation in EBV-mediated oncogenesis. For example, Bcl6 is a zinc-finger transcriptional repressor that functions as a master regulator of B cell development in the germinal center (GC) [15,16]. Frequent dysregulation of Bcl6 expression is involved in various B cell malignancies through disruption of germinal center formation [15,16]. A large number of cellular functions can be modulated by Bcl6 in GC development, including cell survival, cell cycle, DNA damage, and cell differentiation [17,18]. Thus, Bcl6 can be therapeutically targeted by rationally designed inhibitors for treatment of associated lymphomas [19,20]. Furthermore, previous studies showed that EBNA3C can induce the degradation of Bcl6 protein through the ubiquitin–proteasome-dependent signaling pathway, further promoting cell proliferation, and the cell cycle by targeting Bcl2 and cyclin D1 [21]. EBNA3C directly interacts with cyclin D1 and inhibits its ubiquitination [22]. EBNA3C also stabilizes cyclin D2 by suppressing its ubiquitin-dependent degradation to facilitate cell proliferation [23]. Moreover, p21 and p27 are two cyclin-dependent kinase (CDK) inhibitors that block CDK activity in cell cycle regulation [24,25]. Functional loss of p21 or p27 can facilitate the development of human cancers [26]. EBNA3C recruits the E3 ubiquitin ligase SCF^Skp2^ to cyclin A complex and induces SCF^Skp2^-dependent p27 ubiquitination and degradation [27]. EBNA3C also physically interacts with the oncogenic serine/threonine kinase Pim-1 and stabilizes Pim-1 by suppressing its poly-ubiquitination [28]. Overexpression of Pim-1 has been shown to play a role in the progression of hematopoietic malignancies [29]. Further, EBNA3C enhances Pim-1 mediated p21 degradation through the ubiquitin–proteasome pathway, which promotes proliferation of EBV-infected B cells [28].

**Figure 2:**
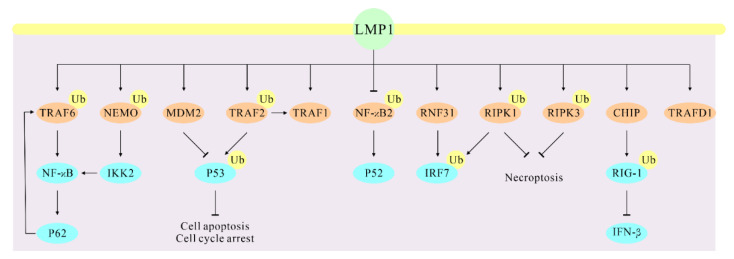
LMP1 regulates the ubiquitination of these direct partners that results in altered cellular signaling of their associated functions. The direct interaction partners of LMP1 are marked with light brown.

EBV latent membrane protein 1 (LMP1) is another essential viral antigen for EBV-mediated transformation, of which LMP1-induced NF-κB activation is necessary for survival of EBV-transformed lymphoblastoid cells [30,31] (Figure 2). NF-κB activation is usually blocked by inhibitors of kappa B (IκBs), and the IκB kinase (IKK) promotes proteasomal degradation of IκB which leads to NF-κB activation [32]. TRAF6 induces IKK activation through K63-linked ubiquitination [33,34]. Moreover, LMP1 activates NF-κB (p65) signaling pathway by inducing TRAF6 poly-ubiquitinated modification in EBV latency, while EBV-encoded BPLF1 interacts with, and deubiquitinates TRAF6 to inhibit the NF-κB signaling pathway during EBV lytic replication [35,36,37]. LMP1 promotes p53 stability by inhibiting K48-linked ubiquitination of p53 mediated by the E3 ligase MDM2, while LMP1 enhances p53 accumulation by inducing K63-linked ubiquitination of p53 that is mediated by the tumor necrosis factor receptor-associated factor 2 (TRAF2), contributing to the suppression of cell apoptosis and cell cycle arrest in EBV latently infected cells [38]. The ubiquitin sensor and adaptor protein SQSTM1/p62 has multiple oncogenic roles during diverse conditions [39,40]. p62 is an autophagy adaptor that contributes to formation of protein aggregates and can also be regulated as a substrate by autophagy [41,42]. Additionally, p62 induces K63-polyubiquitination of TRAF6 to regulate NF-κB activation [43]. During EBV latency, LMP1 activates p62 through NF-κB and AP1, then p62 promotes LMP1-mediated TRAF6 ubiquitination [44]. A deficiency in p62 expression in EBV-transformed B cells can inhibit LMP1-mediated cell proliferation, and suggests p62 as a novel protein in LMP1-induced oncogenic pathways [44].

Different from the canonical activated NF-κB pathway, LMP1 induces degradation of NF-κB p100 subunit through the ubiquitin–proteasome system and promotes translocation of activated p52 together with p65, the RelB NF-κB subunit to the nucleus [45]. This signaling pathway is induced independently of IKKγ/NEMO that is critical for activation of the LMP1-mediated canonical NF-κB pathway, suggesting a novel signaling pathway in LMP1-induced NF-κB activation [45].

LMP1 interacts with RNF31, a critical component of linear ubiquitin assembly complex (LUBAC), and LUBAC is responsible for ubiquitination of NEMO and interferon regulatory factor 7 (IRF7) [46]. Moreover, RNF31 downregulation in EBV-positive cells inhibits LMP1-related downstream genes and suppresses cell proliferation [46]. LMP1-induced IKK2 activation is dependent on NEMO ubiquitination and related to the activation of the downstream canonical NF-κB and JNK signaling pathways [47,48]. LMP1 induces the expression of A20 and IRF7, but A20 negatively regulates IRF7 ubiquitination in EBV latency [49]. LMP1 also stimulates IRF7 activation by promoting ubiquitination of receptor-interacting protein kinase 1 (RIPK1) that is critical for TNF-induced NF-κB activation [50,51,52]. LMP1 enhances K63-linked polyubiquitination of the death domain of kinase RIPK1, but inhibits K63-linked polyubiquitination of RIPK3 through direct interaction with RIPK1 or RIPK3, leading to the suppression of necroptosis [53]. Additionally, the LMP1 TES1/CTAR1 domain can recruit TRAF1 to activate the p38, JNK, ERK, and canonical NF-κB pathways. LMP1 TES1 domain also induces the interaction of TRAF1 and LUBAC, and triggers the attachment of linear (M1)-linked polyubiquitin chains to TRAF1 complexes, both of which are mediated by TRAF2 protein [54]. These findings show that LUBAC-induced linear ubiquitination is crucial for LMP1-medicated NF-κB activation in EBV-infected cells.

A screen using co-immunoprecipitation coupled with mass spectrometry (IP-MS) showed that LMP1 can interact with 19 E3 ligases, including CHIP and TRAFD1 [55]. CHIP directly interacts with RIG-I and is responsible for RIG-I degradation via K48-linked ubiquitination [56]. In addition, LMP1 can inhibit IFN-β expression through promoting RIG-I degradation in nasopharyngeal carcinoma (NPC) cell line C666-1, suggesting that LMP1 may recruit CHIP E3 ligase to degrade RIG-I through the ubiquitin–proteasome system [55]. However, further studies will be required to explore the detailed mechanism by which RIG-I is degraded.

EBV-encoded latent membrane protein 2A (LMP2A) amino-terminal domain can specifically bind to four cellular proteins, including AIP4, WWP2/AIP2, and Nedd4, all of which belong to the Nedd4-like ubiquitin-protein ligase family [57]. LMP2A can recruit these ubiquitin-protein ligases to induce degradation of the downstream Lyn protein tyrosine kinase [57,58]. LMP2A utilizes these Nedd4 family members to trigger the ubiquitination of Lyn and Syk protein tyrosine kinases, which can lead to regulation of LMP2A-mediated B-cell signaling and the maintenance of viral latency [59,60]. In LMP2A^+^ Itch^-/-^ mice, the increased growth of bone marrow B cells demonstrates that Itchy acts as a Nedd4 ubiquitin ligase to negatively regulate LMP2A activity [61]. Besides Itchy, LMP2A is ubiquitinated by the Nedd-family E3 ligases AIP4 and WWP2 [62].

Furthermore, LMP2A enhances MYC expression and suppressed p53-mediated apoptosis in a mouse model [63]. LMP2A can also induce expression of the adaptor protein cyclin-dependent kinase regulatory subunit 1 (Cks1), which degrades the tumor suppressor p27^Kip1^ in a ubiquitin–proteasome dependent manner [63]. Loss of Cks1 results in the prolong of LMP2A-induced lymphomagenesis in mice [63]. Notably, a study using LMP2A and MYC transgenic mice indicated that p27^Kip1^ degradation is required for LMP2A-driven lymphomagenesis [64]. EBV stabilizes β-Catenin in EBV-associated type Ⅲ latency but degrades it in type Ⅰ latency, which involves the function of deubiquitinating enzymes [65]. LMP1 upregulates β-Catenin expression by inhibiting seven in absentia homolog 1 (Siah-1) ubiquitin ligase-mediated ligation in B lymphoma cells [66]. Another study indicated that LMP2A activates PI3K/AKT signaling pathway to stabilize β-Catenin in epithelial cells, but it is not clear whether this is the case in EBV-infected B cells [67]. Moreover, how activated PI3K/AKT signaling is associated with deubiquitinating enzymes is not understood and needs further investigation. LMP2A activates the extracellular signal regulated kinase (ERK) signaling pathway and downregulates levels of the pro-apoptotic protein Bim via proteasomal degradation in EBV-infected cells [68]. Therefore, both LMP1 and LMP2A can interact with cellular ubiquitin ligases to modulate the ubiquitin–proteasome pathway. A study using label-free quantitative proteomics identified many proteins that are regulated by LMP1 and LMP2A [69]. Although they may target distinct cellular factors, they can affect common signaling pathways through recruitment of the ubiquitin degradation pathway [69].

A recent study showed that the EBV-encoded noncoding RNA EBER2 binds to the mRNA of the UCHL1 and recruits its transcription transactivator PU.1 to induce UCHL1 expression. This leads to increased expression of the downstream Aurora kinases and cyclin B1 to further promote cell growth [70]. This suggests that noncoding RNAs can modulate the deubiquitinase to regulate cell growth or cell cycle, but these functions may be related to the specific cell types and the different EBV strains.

## 3. EBV Lytic Antigens Modulate the Activities of the Ubiquitin–Proteasome System for Protein Degradation

A systematic analysis revealed quantitative temporal proteomic profiling that included 8318 host proteins and 69 EBV proteins during EBV lytic replication. These proteins are involved in multiple signaling pathways [71]. Among them, an EBV early protein targets the B cell receptor (BCR) complex for ubiquitin-dependent proteasomal degradation, facilitating cell replication in EBV-infected B cells [71]. EBV-encoded Zta and Rta proteins play a central role in the switch of EBV latency and lytic replication [72,73]. These two critical immediate-early proteins are responsible for expression of all other EBV lytic genes during EBV reactivation [73]. Rta can interact with the E3 ubiquitin ligase TRIM5α in vitro and colocalize in the nucleus during EBV lytic replication [74]. Furthermore, TRIM5α can induce Rta ubiquitination which results in inhibition of EBV lytic progression [74].

To escape the recognition of human T cells, the late lytic BDLF3 protein degrades the major histocompatibility complex (MHC) class I molecules in a ubiquitin–proteasome dependent manner and induces increased internalization and delayed appearance of these MHC molecules on the surface of CD8^+^ T cells [75]. BDLF3 also targets MHC class II molecules of CD4^+^ T cells [75]. The reduced expression of MHC class I and II molecules on human T cells impairs the recognition of EBV late lytic proteins by these T-cells.

The EBV large tegument protein BPLF1 is a known ubiquitin deconjugase that targets the autophagy receptor SQSTM1/p62 (sequestosome 1) in vesicular trafficking and autophagy [76]. BPLF1 directly interacts with p62 and inhibits its ubiquitination [76]. A recent study demonstrated that BPLF1 could target topoisomerase II (TOP2) and stabilize sumoylated TOP2. This results in inhibition of the DNA damage response and etoposide-induced apoptosis [77]. The resistance of etoposide toxicity in EBV-transformed cells is mediated by the expression of tyrosyl-DNA phosphodiesterase 2 (TDP2) that promotes TOP2 releases and DNA repair [77]. BPLF1 can disturb the cellular DNA repair pathway through the deubiquitination of the DNA processivity factor PCNA [78]. Furthermore, BPLF1 directly interacts with the E3 ubiquitin ligase Rad18 and stabilizes Rad18 protein to promote EBV lytic replication and the production of infectious viruses [78]. BPLF1 also induces TRIM25 ubiquitination and inhibits RIG-I ubiquitination to halt the innate anti-viral response [79]. Meanwhile, BPLF1 interacts with and deubiquitinates PCNA to reduce the localization of polymerase η (Pol η) to the nuclear repair foci, leading to the disruption of translesion synthesis [80]. BPLF1 also deubiquitinates EBV ribonucleotide reductase (RR) and reduces its activity in regulation of EBV replication [81].

A functional screen identified the EBV tegument protein BGLF2, which suppresses the host interferon (IFN) signaling pathway [82]. In particular, BGLF2 recruits Cullin 1 E3 ligase to promote STAT2 degradation via K48-linked polyubiquitination. This facilitates EBV primary infection by inhibiting IFN signaling [82]. Another study reported that BGLF2 counteracts type Ⅰ IFN signaling but with a different mechanism than that where BGLF2 interacts with the TYK2 tyrosine kinase to reduce the type Ⅰ IFN signaling [83]. EBV-encoded lytic protein BBRF2 interacts with its partner BSRF1 to tether EBV nucleocapsids, and mechanistically, BBRF2 stabilizes BSRF1 by inhibiting the ubiquitin–proteasome pathway, contributing to augmented EBV infectivity [84,85].

## 4. EBV-Encoded Proteins Can Be Modified by the Ubiquitin–Proteasome System

A study showed that deletion of the Gly-Ala repetitive domain of EBNA1 could enhance its degradation via the ubiquitin–proteasome pathway, implying that Gly-Ala repeats promoted EBNA1 stability [86]. More strikingly, the length of the repeats and the types of degradation signal affect the stability of EBNA1, and these further hamper major histocompatibility complex (MHC) class I-mediated antigen processing and facilitate immune escape [86,87,88,89]. A natural product triptolide, which induces apoptosis and reduces cell proliferation, led to a reduction in EBNA1 expression by inducing the ubiquitin-dependent protein degradation in nasopharyngeal carcinoma (NPC) cells [90]. Using the established lymphoblastoid cell lines (LCLs) stably expressing Flag-HA tagged EBNA3 proteins, the investigators identified distinct interaction complexes of individual EBNA3 proteins [91]. They found that EBNA3 proteins can interact with USP46 deubiquitinating enzyme (DUB) as well as its associated chaperones WDR48 and WDR20 [91]. Although the DUB complex is recruited by EBNA3 protein, more evidence is needed to explore how these complexes target the specific substrates. Besides, LMP1 itself can be ubiquitinated through the ubiquitin–proteasome pathway [92]. Ribosomal protein S27a (RPS27a) interacts with LMP1 and enhances LMP1 expression via inhibition of its proteasomal degradation in EBV-infected cells [93].

The immediate-early protein Zta can also be modified by ubiquitination. This is facilitated by targeting four lysine residues on the Zta protein [94]. This type of modification inhibits the stability of Zta, and therefore, the following viral lytic replication, demonstrating the important function of ubiquitination on regulating the EBV life cycle [94]. RNF4, a RING-domain-containing ubiquitin E3 ligase, can directly target Rta and induce the ubiquitination of SUMO-2-conjugated Rta [95]. The mutation of lysine residues on Rta impairs its sumoylation and decreases RNF-4 mediated Rta ubiquitination. Therefore, it suggests that RNF4 acts as a SUMO-targeted ubiquitin E3 ligase of Rta to modulate EBV lytic replication [95].

After screening a kinase inhibitor library, one study found that cyclin-dependent kinase (CDK) inhibitors can induce degradation of the viral lytic protein BDLF4, which is important for EBV lytic replication, and progeny production [96,97]. CDK2 complexes phosphorylate BDLF4 at threonine 91 to protect BDLF4 from ubiquitin-dependent degradation [96]. EBV-encoded BFRF1 protein regulates the nuclear envelope (NE)-derived vesicles by recruiting the Alix protein, which is associated with cellular endosomal sorting complex required for the transport (ESCRT) machinery [98]. BFRF1 ubiquitination is mediated by the ubiquitin ligase Itch and modulates the formation of BFRF1-driven NE vesicles [98]. Interestingly, Itch is associated with both BFRF1 and Alix proteins, suggesting that these molecular players interact with each other to control BFRF1-induced NE vesicles, and EBV maturation [98].

EBV envelop glycoprotein B (gB) is a key protein as a member of the fusion machinery required for viral entry into B cells and epithelial cells [99]. The E3 ligase F-box only protein 2 (FBXO2) was identified due to its ability to recognize N-glycosylated gB, and induced its degradation through the ubiquitin–proteasome pathway. This resulted in the suppression of EBV infectivity [100]. These findings represent a new host defense mechanism strategy against EBV infection.

## 5. Targeting EBV-Associated Oncogenesis with Proteasome Inhibitors

The proteasome in the ubiquitin–proteasome pathway has been identified as a therapeutic target for treatment of many cancers. More specifically, proteasome inhibitors have also been used for targeting EBV-associated diseases. Bortezomib (Velcade) is the first FDA-approved reversible proteasome inhibitor [101,102]. Bortezomib suppressed the growth of EBV-positive Burkitt’s lymphoma in a murine xenograft model. Moreover, the tumor growth was almost completely halted after treatment with Bortezomib followed by the nucleoside analogue [^131^I]2′-fluoro-2′-deoxy-beta-D-5-iodouracil-arabinofuranoside ([^131^I]FIAU) [103,104]. Further studies showed that Bortezomib treatment enhances the binding of CCAAT/enhancer-binding proteinβ (C/EBPβ) to the Zta promoter and induced Zta-mediated EBV lytic replication [105]. Ixazomib (Ninlaro) is another FDA-approved oral proteasome inhibitor [106,107]. One study indicated that Ixazomib promoted accumulation of polyubiquitinated proteins and induces cell cycle arrest and apoptosis in EBV-associated B-lymphoblastoid cells [108]. Both Bortezomib and Ixazomib have a similar structure and can inhibit the β1 caspase-like and β2 trypsin-like subunits of the 20S proteasome [106,109]. Epoxomicin is a natural product that can specifically target the 20S proteasome and function as a selective and irreversible proteasome inhibitor [110,111]. However, Epoxomicin has poor drug-like features including the labile epoxy ketone pharmacophore, which restricts its development as a potential proteasome inhibitor [112,113]. Furthermore, Carfilzomib (Kyprolis), a derivative of Epoxomicin, becomes another FDA-approved irreversible proteasome inhibitor, which can more preferentially inhibit the chymotrypsin-like subunit β5 of 20S proteasome [114,115,116]. Although Carfilzomib exhibits improved efficacy and safety over Bortezomib, its effects on EBV-associated lymphomas remain unknown [117]. Other proteasome inhibitors, such as Marizomib, Oprozomib, and Delanzomib are still in clinical trials [118]. Moreover, ubiquitin C-terminal hydrolase L1 (UCHL1) was highly expressed after more than 30 days post-infection during establishment of EBV-transformed LCLs, and may be associated with EBER regulation [9,70]. A selected small-molecule inhibitor targeting the deubiquitinating enzyme (DUB) UCHL1, LDN-57444 or its soluble form LDN-Pox, was shown to suppress the motility of EBV-positive nasopharyngeal cells [119]. This suggests that DUB can be a potential therapeutic target for treating cancers. Although these drugs are recognized as proteasome inhibitors, their non-proteasome targets still need further investigation.

A high throughput screening of small molecule compounds identified five tetrahydrocarboline derivatives that effectively reactivated the lytic cycle through induction of the transcription activity of EBV immediate-early Zta gene [120]. Among these compounds, C60 consistently stimulated EBV lytic reactivation and can synergize with Ganciclovir (GCV) to selectively eliminate EBV-positive tumor cells [120]. A following biochemical affinity purification assay showed that C60 can directly target the Cullin exchange factor CAND1 [121]. Further, C60 disturbs the association of CAND1 with Cullin 1 and accumulates the global ubiquitylated substrates [121]. This stabilizes the EBV Zta protein by regulating the ubiquitin-dependent proteasome pathway, which leads to EBV lytic reactivation from latency [121].

## 6. Conclusions and Perspectives

The ubiquitin–proteasome system (UPS) is central to the regulation of the stability of cellular factors as well as their related signaling pathways. EBV has developed multiple strategies that manipulate the ubiquitin system to induce oncogenesis or escape immune response in EBV-infected cells. Therefore, the ubiquitin–proteasome system becomes an important therapeutic target for development of interventions to treat EBV-associated diseases. Several proteasome inhibitors have been approved, or in clinical trials, but the toxicity and resistance of these inhibitors restrict their wide application because of the accumulation of ubiquitinated proteins [122]. The combination of proteasome inhibitors and HDAC inhibitors or other immunotherapies as a future direction has the potential to be used in EBV-associated cancers [123,124,125]. Furthermore, the development of novel EBV-specific therapeutic agents, which can modulate the interactions of viral antigens and their cellular binding partners, or target viral antigens-related E3 ligase, will offer novel strategies against EBV-associated cancers.

## Figures and Tables

**Table 1:** The interaction of EBV antigens and cellular factors targeted for protein degradation. The cellular proteins that directly interact with the indicated EBV antigens are listed, which shows that both EBV latent and lytic genes are involved in regulating degradation of these substrates.

EBV Life Cycle	EBV Antigens	Cellular Factors
Latent cycle	EBNA1	USP7 CK2 PRMT5
	EBNA3C	Bcl6 Cyclin D1 Cyclin D2 Skp2 Pim-1
	LMP1	RIPK1 RIPK3 P53 TRAF1 TRAF6 NF-κB2 p100 RNF31 IRF7 CHIP TRAFD1
	LMP2A	AIP4 WWP2 Nedd4 Siah-1
Lytic cycle	Rta	TRIM5α
	BDLF3	MHC-I MHC-II
	BPLF1	P62 TOP2 TRIM25 PCNA Rad18 RR
	BGLF2	Cullin 1 TYK2

## References

[1] Glickman M.H., Ciechanover A. (2002). The ubiquitin–proteasome proteolytic pathway: Destruction for the sake of construction. Physiol. Rev..

[2] Komander D., Rape M. (2012). The ubiquitin code. Annu. Rev. Biochem..

[3] Shi D., Grossman S.R. (2010). Ubiquitin becomes ubiquitous in cancer: Emerging roles of ubiquitin ligases and deubiquitinases in tumorigenesis and as therapeutic targets. Cancer. Biol. Ther..

[4] Weissman A.M. (2001). Themes and variations on ubiquitylation. Nat. Rev. Mol. Cell Biol..

[5] Kutok J.L., Wang F. (2006). Spectrum of Epstein–Barr virus-associated diseases. Annu. Rev. Pathol..

[6] Pei Y., Wong J.H., Robertson E.S. (2020). Herpesvirus Epigenetic Reprogramming and Oncogenesis. Annu. Rev. Virol..

[7] Luo H. (2016). Interplay between the virus and the ubiquitin–proteasome system: Molecular mechanism of viral pathogenesis. Curr. Opin. Virol..

[8] Hui K.F., Tam K.P., Chiang A.K.S. (2017). Therapeutic Strategies against Epstein–Barr Virus-Associated Cancers Using Proteasome Inhibitors. Viruses.

[9] Ovaa H., Kessler B.M., Rolen U., Galardy P.J., Ploegh H.L., Masucci M.G. (2004). Activity-based ubiquitin-specific protease (USP) profiling of virus-infected and malignant human cells. Proc. Natl. Acad. Sci. USA.

[10] Holowaty M.N., Zeghouf M., Wu H., Tellam J., Athanasopoulos V., Greenblatt J., Frappier L. (2003). Protein profiling with Epstein–Barr nuclear antigen-1 reveals an interaction with the herpesvirus-associated ubiquitin-specific protease HAUSP/USP7. J. Biol. Chem..

[11] Sivachandran N., Cao J.Y., Frappier L. (2010). Epstein–Barr virus nuclear antigen 1 Hijacks the host kinase CK2 to disrupt PML nuclear bodies. J. Virol..

[12] Saridakis V., Sheng Y., Sarkari F., Holowaty M.N., Shire K., Nguyen T., Zhang R.G., Liao J., Lee W., Edwards A.M. (2005). Structure of the p53 binding domain of HAUSP/USP7 bound to Epstein–Barr nuclear antigen 1 implications for EBV-mediated immortalization. Mol. Cell..

[13] Holowaty M.N., Frappier L. (2004). HAUSP/USP7 as an Epstein–Barr virus target. Biochem. Soc. Trans..

[14] Tomkinson B., Robertson E., Kieff E. (1993). Epstein–Barr virus nuclear proteins EBNA-3A and EBNA-3C are essential for B-lymphocyte growth transformation. J. Virol..

[15] Migliazza A., Martinotti S., Chen W., Fusco C., Ye B.H., Knowles D.M., Offit K., Chaganti R.S., Dalla-Favera R. (1995). Frequent somatic hypermutation of the 5’ noncoding region of the BCL6 gene in B-cell lymphoma. Proc. Natl. Acad. Sci. USA.

[16] Gaidano G., Carbone A., Pastore C., Capello D., Migliazza A., Gloghini A., Roncella S., Ferrarini M., Saglio G., Dalla-Favera R. (1997). Frequent mutation of the 5’ noncoding region of the BCL-6 gene in acquired immunodeficiency syndrome-related non-Hodgkin’s lymphomas. Blood.

[17] Basso K., Saito M., Sumazin P., Margolin A.A., Wang K., Lim W.K., Kitagawa Y., Schneider C., Alvarez M.J., Califano A. (2010). Integrated biochemical and computational approach identifies BCL6 direct target genes controlling multiple pathways in normal germinal center B cells. Blood.

[18] Basso K., Dalla-Favera R. (2010). BCL6: Master regulator of the germinal center reaction and key oncogene in B cell lymphomagenesis. Adv. Immunol..

[19] Cerchietti L.C., Ghetu A.F., Zhu X., Da Silva G.F., Zhong S., Matthews M., Bunting K.L., Polo J.M., Fares C., Arrowsmith C.H. (2010). A small-molecule inhibitor of BCL6 kills DLBCL cells in vitro and in vivo. Cancer Cell.

[20] Cardenas M.G., Yu W., Beguelin W., Teater M.R., Geng H., Goldstein R.L., Oswald E., Hatzi K., Yang S.N., Cohen J. (2016). Rationally designed BCL6 inhibitors target activated B cell diffuse large B cell lymphoma. J. Clin. Investig..

[21] Pei Y., Banerjee S., Jha H.C., Sun Z., Robertson E.S. (2017). An essential EBV latent antigen 3C binds Bcl6 for targeted degradation and cell proliferation. PLoS Pathog..

[22] Saha A., Halder S., Upadhyay S.K., Lu J., Kumar P., Murakami M., Cai Q., Robertson E.S. (2011). Epstein–Barr virus nuclear antigen 3C facilitates G1-S transition by stabilizing and enhancing the function of cyclin D1. PLoS Pathog..

[23] Pei Y., Singh R.K., Shukla S.K., Lang F., Zhang S., Robertson E.S. (2018). Epstein–Barr Virus Nuclear Antigen 3C Facilitates Cell Proliferation by Regulating Cyclin D2. J. Virol..

[24] Xiong Y., Hannon G.J., Zhang H., Casso D., Kobayashi R., Beach D. (1993). p21 is a universal inhibitor of cyclin kinases. Nature.

[25] Toyoshima H., Hunter T. (1994). p27, a novel inhibitor of G1 cyclin-Cdk protein kinase activity, is related to p21. Cell.

[26] Abukhdeir A.M., Park B.H. (2008). P21 and p27: Roles in carcinogenesis and drug resistance. Expert Rev. Mol. Med..

[27] Knight J.S., Sharma N., Robertson E.S. (2005). SCFSkp2 complex targeted by Epstein–Barr virus essential nuclear antigen. Mol. Cell. Biol..

[28] Banerjee S., Lu J., Cai Q., Sun Z., Jha H.C., Robertson E.S. (2014). EBNA3C augments Pim-1 mediated phosphorylation and degradation of p21 to promote B-cell proliferation. PLoS Pathog..

[29] Hu X.F., Li J., Vandervalk S., Wang Z., Magnuson N.S., Xing P.X. (2009). PIM-1-specific mAb suppresses human and mouse tumor growth by decreasing PIM-1 levels, reducing Akt phosphorylation, and activating apoptosis. J. Clin. Investig..

[30] McFarland E.D.C., Izumi K.M., Mosialos G. (1999). Epstein–barr virus transformation: Involvement of latent membrane protein 1-mediated activation of NF-kappaB. Oncogene.

[31] Cahir-McFarland E.D., Davidson D.M., Schauer S.L., Duong J., Kieff E. (2000). NF-kappa B inhibition causes spontaneous apoptosis in Epstein–Barr virus-transformed lymphoblastoid cells. Proc. Natl. Acad. Sci. USA.

[32] Hayden M.S., Ghosh S. (2004). Signaling to NF-kappaB. Genes Dev..

[33] Deng L., Wang C., Spencer E., Yang L., Braun A., You J., Slaughter C., Pickart C., Chen Z.J. (2000). Activation of the IkappaB kinase complex by TRAF6 requires a dimeric ubiquitin-conjugating enzyme complex and a unique polyubiquitin chain. Cell.

[34] Wang C., Deng L., Hong M., Akkaraju G.R., Inoue J., Chen Z.J. (2001). TAK1 is a ubiquitin-dependent kinase of MKK and IKK. Nature.

[35] Saito S., Murata T., Kanda T., Isomura H., Narita Y., Sugimoto A., Kawashima D., Tsurumi T. (2013). Epstein–Barr virus deubiquitinase downregulates TRAF6-mediated NF-kappaB signaling during productive replication. J. Virol..

[36] Schultheiss U., Puschner S., Kremmer E., Mak T.W., Engelmann H., Hammerschmidt W., Kieser A. (2001). TRAF6 is a critical mediator of signal transduction by the viral oncogene latent membrane protein 1. EMBO J..

[37] Arcipowski K.M., Stunz L.L., Graham J.P., Kraus Z.J., Vanden Bush T.J., Bishop G.A. (2011). Molecular mechanisms of TNFR-associated factor 6 (TRAF6) utilization by the oncogenic viral mimic of CD40, latent membrane protein 1 (LMP1). J. Biol. Chem..

[38] Li L., Li W., Xiao L., Xu J., Chen X., Tang M., Dong Z., Tao Q., Cao Y. (2012). Viral oncoprotein LMP1 disrupts p53-induced cell cycle arrest and apoptosis through modulating K63-linked ubiquitination of p53. Cell Cycle.

[39] Thompson H.G., Harris J.W., Wold B.J., Lin F., Brody J.P. (2003). p62 overexpression in breast tumors and regulation by prostate-derived Ets factor in breast cancer cells. Oncogene.

[40] Duran A., Linares J.F., Galvez A.S., Wikenheiser K., Flores J.M., Diaz-Meco M.T., Moscat J. (2008). The signaling adaptor p62 is an important NF-kappaB mediator in tumorigenesis. Cancer Cell.

[41] Komatsu M., Waguri S., Koike M., Sou Y.S., Ueno T., Hara T., Mizushima N., Iwata J., Ezaki J., Murata S. (2007). Homeostatic levels of p62 control cytoplasmic inclusion body formation in autophagy-deficient mice. Cell.

[42] Shin J. (1998). P62 and the sequestosome, a novel mechanism for protein metabolism. Arch. Pharm. Res..

[43] Wooten M.W., Geetha T., Seibenhener M.L., Babu J.R., Diaz-Meco M.T., Moscat J. (2005). The p62 scaffold regulates nerve growth factor-induced NF-kappaB activation by influencing TRAF6 polyubiquitination. J. Biol. Chem..

[44] Wang L., Howell M.E.A., Sparks-Wallace A., Zhao J., Hensley C.R., Nicksic C.A., Horne S.R., Mohr K.B., Moorman J.P., Yao Z.Q. (2021). The Ubiquitin Sensor and Adaptor Protein p62 Mediates Signal Transduction of a Viral Oncogenic Pathway. mBio.

[45] Eliopoulos A.G., Caamano J.H., Flavell J., Reynolds G.M., Murray P.G., Poyet J.L., Young L.S. (2003). Epstein–Barr virus-encoded latent infection membrane protein 1 regulates the processing of p100 NF-kappaB2 to p52 via an IKKgamma/NEMO-independent signalling pathway. Oncogene.

[46] Wang L., Wang Y., Zhao J., Ren J., Hall K.H., Moorman J.P., Yao Z.Q., Ning S. (2017). The Linear Ubiquitin Assembly Complex Modulates Latent Membrane Protein 1 Activation of NF-kappaB and Interferon Regulatory Factor 7. J. Virol..

[47] Hinz M., Scheidereit C. (2014). The IkappaB kinase complex in NF-kappaB regulation and beyond. EMBO Rep..

[48] Voigt S., Sterz K.R., Giehler F., Mohr A.W., Wilson J.B., Moosmann A., Kieser A. (2020). A central role of IKK2 and TPL2 in JNK activation and viral B-cell transformation. Nat. Commun..

[49] Ning S., Pagano J.S. (2010). The A20 deubiquitinase activity negatively regulates LMP1 activation of IRF7. J. Virol..

[50] Hsu H., Huang J., Shu H.B., Baichwal V., Goeddel D.V. (1996). TNF-dependent recruitment of the protein kinase RIP to the TNF receptor-1 signaling complex. Immunity.

[51] Kelliher M.A., Grimm S., Ishida Y., Kuo F., Stanger B.Z., Leder P. (1998). The death domain kinase RIP mediates the TNF-induced NF-kappaB signal. Immunity.

[52] Huye L.E., Ning S., Kelliher M., Pagano J.S. (2007). Interferon regulatory factor 7 is activated by a viral oncoprotein through RIP-dependent ubiquitination. Mol. Cell. Biol..

[53] Liu X., Li Y., Peng S., Yu X., Li W., Shi F., Luo X., Tang M., Tan Z., Bode A.M. (2018). Epstein–Barr virus encoded latent membrane protein 1 suppresses necroptosis through targeting RIPK1/3 ubiquitination. Cell Death Dis..

[54] Greenfeld H., Takasaki K., Walsh M.J., Ersing I., Bernhardt K., Ma Y., Fu B., Ashbaugh C.W., Cabo J., Mollo S.B. (2015). TRAF1 Coordinates Polyubiquitin Signaling to Enhance Epstein–Barr Virus LMP1-Mediated Growth and Survival Pathway Activation. PLoS Pathog..

[55] Xu C., Sun L., Liu W., Duan Z. (2018). Latent Membrane Protein 1 of Epstein–Barr Virus Promotes RIG-I Degradation Mediated by Proteasome Pathway. Front. Immunol..

[56] Zhao K., Zhang Q., Li X., Zhao D., Liu Y., Shen Q., Yang M., Wang C., Li N., Cao X. (2016). Cytoplasmic STAT4 Promotes Antiviral Type I IFN Production by Blocking CHIP-Mediated Degradation of RIG-I. J. Immunol..

[57] Ikeda M., Ikeda A., Longan L.C., Longnecker R. (2000). The Epstein–Barr virus latent membrane protein 2A PY motif recruits WW domain-containing ubiquitin-protein ligases. Virology.

[58] Fruehling S., Swart R., Dolwick K.M., Kremmer E., Longnecker R. (1998). Tyrosine 112 of latent membrane protein 2A is essential for protein tyrosine kinase loading and regulation of Epstein–Barr virus latency. J. Virol..

[59] Winberg G., Matskova L., Chen F., Plant P., Rotin D., Gish G., Ingham R., Ernberg I., Pawson T. (2000). Latent membrane protein 2A of Epstein–Barr virus binds WW domain E3 protein-ubiquitin ligases that ubiquitinate B-cell tyrosine kinases. Mol. Cell. Biol..

[60] Ikeda M., Ikeda A., Longnecker R. (2001). PY motifs of Epstein–Barr virus LMP2A regulate protein stability and phosphorylation of LMP2A-associated proteins. J. Virol..

[61] Ikeda A., Caldwell R.G., Longnecker R., Ikeda M. (2003). Itchy, a Nedd4 ubiquitin ligase, downregulates latent membrane protein 2A activity in B-cell signaling. J. Virol..

[62] Ikeda M., Ikeda A., Longnecker R. (2002). Lysine-independent ubiquitination of Epstein–Barr virus LMP2A. Virology.

[63] Fish K., Sora R.P., Schaller S.J., Longnecker R., Ikeda M. (2017). EBV latent membrane protein 2A orchestrates p27(kip1) degradation via Cks1 to accelerate MYC-driven lymphoma in mice. Blood.

[64] Sora R.P., Ikeda M., Longnecker R. (2019). Two Pathways of p27(Kip1) Degradation Are Required for Murine Lymphoma Driven by Myc and EBV Latent Membrane Protein 2A. mBio.

[65] Shackelford J., Maier C., Pagano J.S. (2003). Epstein–Barr virus activates beta-catenin in type III latently infected B lymphocyte lines: Association with deubiquitinating enzymes. Proc. Natl. Acad. Sci. USA.

[66] Jang K.L., Shackelford J., Seo S.Y., Pagano J.S. (2005). Up-regulation of beta-catenin by a viral oncogene correlates with inhibition of the seven in absentia homolog 1 in B lymphoma cells. Proc. Natl. Acad. Sci. USA.

[67] Morrison J.A., Klingelhutz A.J., Raab-Traub N. (2003). Epstein–Barr virus latent membrane protein 2A activates beta-catenin signaling in epithelial cells. J. Virol..

[68] Iwakiri D., Minamitani T., Samanta M. (2013). Epstein–Barr virus latent membrane protein 2A contributes to anoikis resistance through ERK activation. J. Virol..

[69] DeKroon R.M., Gunawardena H.P., Edwards R., Raab-Traub N. (2018). Global Proteomic Changes Induced by the Epstein–Barr Virus Oncoproteins Latent Membrane Protein 1 and 2A. mBio.

[70] Li Z., Baccianti F., Delecluse S., Tsai M.H., Shumilov A., Cheng X., Ma S., Hoffmann I., Poirey R., Delecluse H.J. (2021). The Epstein–Barr virus noncoding RNA EBER2 transactivates the UCHL1 deubiquitinase to accelerate cell growth. Proc. Natl. Acad. Sci. USA.

[71] Ersing I., Nobre L., Wang L.W., Soday L., Ma Y., Paulo J.A., Narita Y., Ashbaugh C.W., Jiang C., Grayson N.E. (2017). A Temporal Proteomic Map of Epstein–Barr Virus Lytic Replication in B Cells. Cell Rep..

[72] Feederle R., Kost M., Baumann M., Janz A., Drouet E., Hammerschmidt W., Delecluse H.J. (2000). The Epstein–Barr virus lytic program is controlled by the co-operative functions of two transactivators. EMBO J..

[73] McKenzie J., El-Guindy A. (2015). Epstein–Barr Virus Lytic Cycle Reactivation. Curr. Top MicroBiol. Immunol..

[74] Huang H.H., Chen C.S., Wang W.H., Hsu S.W., Tsai H.H., Liu S.T., Chang L.K. (2016). TRIM5alpha Promotes Ubiquitination of Rta from Epstein–Barr Virus to Attenuate Lytic Progression. Front. Microbiol..

[75] Quinn L.L., Williams L.R., White C., Forrest C., Zuo J., Rowe M. (2016). The Missing Link in Epstein–Barr Virus Immune Evasion: The BDLF3 Gene Induces Ubiquitination and Downregulation of Major Histocompatibility Complex Class I (MHC-I) and MHC-II. J. Virol..

[76] Yla-Anttila P., Gupta S., Masucci M.G. (2021). The Epstein–Barr virus deubiquitinase BPLF1 targets SQSTM1/p62 to inhibit selective autophagy. Autophagy.

[77] Li J., Nagy N., Liu J., Gupta S., Frisan T., Hennig T., Cameron D.P., Baranello L., Masucci M.G. (2021). The Epstein–Barr virus deubiquitinating enzyme BPLF1 regulates the activity of topoisomerase II during productive infection. PLoS Pathog..

[78] Kumar R., Whitehurst C.B., Pagano J.S. (2014). The Rad6/18 ubiquitin complex interacts with the Epstein–Barr virus deubiquitinating enzyme, BPLF1, and contributes to virus infectivity. J. Virol..

[79] Gupta S., Yla-Anttila P., Callegari S., Tsai M.H., Delecluse H.J., Masucci M.G. (2018). Herpesvirus deconjugases inhibit the IFN response by promoting TRIM25 autoubiquitination and functional inactivation of the RIG-I signalosome. PLoS Pathog..

[80] Whitehurst C.B., Vaziri C., Shackelford J., Pagano J.S. (2012). Epstein–Barr virus BPLF1 deubiquitinates PCNA and attenuates polymerase eta recruitment to DNA damage sites. J. Virol..

[81] Whitehurst C.B., Ning S., Bentz G.L., Dufour F., Gershburg E., Shackelford J., Langelier Y., Pagano J.S. (2009). The Epstein–Barr virus (EBV) deubiquitinating enzyme BPLF1 reduces EBV ribonucleotide reductase activity. J. Virol..

[82] Jangra S., Bharti A., Lui W.Y., Chaudhary V., Botelho M.G., Yuen K.S., Jin D.Y. (2021). Suppression of JAK-STAT Signaling by Epstein–Barr Virus Tegument Protein BGLF2 through Recruitment of SHP1 Phosphatase and Promotion of STAT2 Degradation. J. Virol..

[83] Liu X., Sadaoka T., Krogmann T., Cohen J.I. (2020). Epstein–Barr Virus (EBV) Tegument Protein BGLF2 Suppresses Type I Interferon Signaling To Promote EBV Reactivation. J. Virol..

[84] Masud H., Yanagi Y., Watanabe T., Sato Y., Kimura H., Murata T. (2019). Epstein–Barr Virus BBRF2 Is Required for Maximum Infectivity. Microorganisms.

[85] He H.P., Luo M., Cao Y.L., Lin Y.X., Zhang H., Zhang X., Ou J.Y., Yu B., Chen X., Xu M. (2020). Structure of Epstein–Barr virus tegument protein complex BBRF2-BSRF1 reveals its potential role in viral envelopment. Nat. Commun..

[86] Levitskaya J., Sharipo A., Leonchiks A., Ciechanover A., Masucci M.G. (1997). Inhibition of ubiquitin/proteasome-dependent protein degradation by the Gly-Ala repeat domain of the Epstein–Barr virus nuclear antigen 1. Proc. Natl. Acad. Sci. USA.

[87] Levitskaya J., Coram M., Levitsky V., Imreh S., Steigerwald-Mullen P.M., Klein G., Kurilla M.G., Masucci M.G. (1995). Inhibition of antigen processing by the internal repeat region of the Epstein–Barr virus nuclear antigen-1. Nature.

[88] Sharipo A., Imreh M., Leonchiks A., Imreh S., Masucci M.G. (1998). A minimal glycine-alanine repeat prevents the interaction of ubiquitinated I kappaB alpha with the proteasome: A new mechanism for selective inhibition of proteolysis. Nat. Med..

[89] Dantuma N.P., Heessen S., Lindsten K., Jellne M., Masucci M.G. (2000). Inhibition of proteasomal degradation by the gly-Ala repeat of Epstein–Barr virus is influenced by the length of the repeat and the strength of the degradation signal. Proc. Natl. Acad. Sci. USA.

[90] Zhou H., Liu Y., Wang C., Liu L., Wang H., Zhang Y., Long C., Sun X. (2018). Triptolide inhibits Epstein–Barr nuclear antigen 1 expression by increasing sensitivity of mitochondria apoptosis of nasopharyngeal carcinoma cells. J. Exp. Clin. Cancer Res..

[91] Ohashi M., Holthaus A.M., Calderwood M.A., Lai C.Y., Krastins B., Sarracino D., Johannsen E. (2015). The EBNA3 family of Epstein–Barr virus nuclear proteins associates with the USP46/USP12 deubiquitination complexes to regulate lymphoblastoid cell line growth. PLoS Pathog..

[92] Aviel S., Winberg G., Massucci M., Ciechanover A. (2000). Degradation of the Epstein–barr virus latent membrane protein 1 (LMP1) by the ubiquitin–proteasome pathway. Targeting via ubiquitination of the N-terminal residue. J. Biol. Chem..

[93] Hong S.W., Kim S.M., Jin D.H., Kim Y.S., Hur D.Y. (2017). RPS27a enhances EBV-encoded LMP1-mediated proliferation and invasion by stabilizing of LMP1. BioChem. Biophys. Res. Commun..

[94] Zhao M., Nanbo A., Becnel D., Qin Z., Morris G.F., Li L., Lin Z. (2020). Ubiquitin Modification of the Epstein–Barr Virus Immediate Early Transactivator Zta. J. Virol..

[95] Yang Y.C., Yoshikai Y., Hsu S.W., Saitoh H., Chang L.K. (2013). Role of RNF4 in the ubiquitination of Rta of Epstein–Barr virus. J. Biol. Chem..

[96] Sato Y., Watanabe T., Suzuki C., Abe Y., Masud H., Inagaki T., Yoshida M., Suzuki T., Goshima F., Adachi J. (2019). S-Like-Phase Cyclin-Dependent Kinases Stabilize the Epstein–Barr Virus BDLF4 Protein To Temporally Control Late Gene Transcription. J. Virol..

[97] Watanabe T., Narita Y., Yoshida M., Sato Y., Goshima F., Kimura H., Murata T. (2015). The Epstein–Barr Virus BDLF4 Gene Is Required for Efficient Expression of Viral Late Lytic Genes. J. Virol..

[98] Lee C.P., Liu G.T., Kung H.N., Liu P.T., Liao Y.T., Chow L.P., Chang L.S., Chang Y.H., Chang C.W., Shu W.C. (2016). The Ubiquitin Ligase Itch and Ubiquitination Regulate BFRF1-Mediated Nuclear Envelope Modification for Epstein–Barr Virus Maturation. J. Virol..

[99] Spear P.G., Longnecker R. (2003). Herpesvirus entry: An update. J. Virol..

[100] Zhang H.J., Tian J., Qi X.K., Xiang T., He G.P., Zhang H., Yu X., Zhang X., Zhao B., Feng Q.S. (2018). Epstein–Barr virus activates F-box protein FBXO2 to limit viral infectivity by targeting glycoprotein B for degradation. PLoS Pathog..

[101] Richardson P.G., Mitsiades C., Hideshima T., Anderson K.C. (2006). Bortezomib: Proteasome inhibition as an effective anticancer therapy. Annu. Rev. Med..

[102] Kane R.C., Farrell A.T., Sridhara R., Pazdur R. (2006). United States Food and Drug Administration approval summary: Bortezomib for the treatment of progressive multiple myeloma after one prior therapy. Clin. Cancer Res.

[103] Fu D.X., Tanhehco Y.C., Chen J., Foss C.A., Fox J.J., Lemas V., Chong J.M., Ambinder R.F., Pomper M.G. (2007). Virus-associated tumor imaging by induction of viral gene expression. Clin. Cancer Res..

[104] Fu D.X., Tanhehco Y., Chen J., Foss C.A., Fox J.J., Chong J.M., Hobbs R.F., Fukayama M., Sgouros G., Kowalski J. (2008). Bortezomib-induced enzyme-targeted radiation therapy in herpesvirus-associated tumors. Nat. Med..

[105] Shirley C.M., Chen J., Shamay M., Li H., Zahnow C.A., Hayward S.D., Ambinder R.F. (2011). Bortezomib induction of C/EBPbeta mediates Epstein–Barr virus lytic activation in Burkitt lymphoma. Blood.

[106] Muz B., Ghazarian R.N., Ou M., Luderer M.J., Kusdono H.D., Azab A.K. (2016). Spotlight on ixazomib: Potential in the treatment of multiple myeloma. Drug Des. Dev. Ther..

[107] Shirley M. (2016). Ixazomib: First Global Approval. Drugs.

[108] Ganguly S., Kuravi S., Alleboina S., Mudduluru G., Jensen R.A., McGuirk J.P., Balusu R. (2019). Targeted Therapy for EBV-Associated B-cell Neoplasms. Mol. Cancer Res..

[109] Kupperman E., Lee E.C., Cao Y., Bannerman B., Fitzgerald M., Berger A., Yu J., Yang Y., Hales P., Bruzzese F. (2010). Evaluation of the proteasome inhibitor MLN9708 in preclinical models of human cancer. Cancer Res..

[110] Meng L., Mohan R., Kwok B.H., Elofsson M., Sin N., Crews C.M. (1999). Epoxomicin, a potent and selective proteasome inhibitor, exhibits in vivo antiinflammatory activity. Proc. Natl. Acad. Sci. USA.

[111] Hanada M., Sugawara K., Kaneta K., Toda S., Nishiyama Y., Tomita K., Yamamoto H., Konishi M., Oki T. (1992). Epoxomicin, a new antitumor agent of microbial origin. J. Antibiot..

[112] Kim K.B., Crews C.M. (2013). From epoxomicin to carfilzomib: Chemistry, biology, and medical outcomes. Nat. Prod. Rep..

[113] Myung J., Kim K.B., Crews C.M. (2001). The ubiquitin–proteasome pathway and proteasome inhibitors. Med. Res. Rev..

[114] Harshbarger W., Miller C., Diedrich C., Sacchettini J. (2015). Crystal structure of the human 20S proteasome in complex with carfilzomib. Structure.

[115] Jager S., Groll M., Huber R., Wolf D.H., Heinemeyer W. (1999). Proteasome beta-type subunits: Unequal roles of propeptides in core particle maturation and a hierarchy of active site function. J. Mol. Biol..

[116] Herndon T.M., Deisseroth A., Kaminskas E., Kane R.C., Koti K.M., Rothmann M.D., Habtemariam B., Bullock J., Bray J.D., Hawes J. (2013). U.s. Food and Drug Administration approval: Carfilzomib for the treatment of multiple myeloma. Clin. Cancer Res..

[117] Vij R., Siegel D.S., Jagannath S., Jakubowiak A.J., Stewart A.K., McDonagh K., Bahlis N., Belch A., Kunkel L.A., Wear S. (2012). An open-label, single-arm, phase 2 study of single-agent carfilzomib in patients with relapsed and/or refractory multiple myeloma who have been previously treated with bortezomib. Br. J. Haematol..

[118] Fricker L.D. (2020). Proteasome Inhibitor Drugs. Annu. Rev. Pharmacol. Toxicol..

[119] Kobayashi E., Hwang D., Bheda-Malge A., Whitehurst C.B., Kabanov A.V., Kondo S., Aga M., Yoshizaki T., Pagano J.S., Sokolsky M. (2019). Inhibition of UCH-L1 Deubiquitinating Activity with Two Forms of LDN-57444 Has Anti-Invasive Effects in Metastatic Carcinoma Cells. Int. J. Mol. Sci..

[120] Tikhmyanova N., Schultz D.C., Lee T., Salvino J.M., Lieberman P.M. (2014). Identification of a new class of small molecules that efficiently reactivate latent Epstein–Barr Virus. ACS Chem. Biol..

[121] Tikhmyanova N., Tutton S., Martin K.A., Lu F., Kossenkov A.V., Paparoidamis N., Kenney S., Salvino J.M., Lieberman P.M. (2017). Small molecule perturbation of the CAND1-Cullin1-ubiquitin cycle stabilizes p53 and triggers Epstein–Barr virus reactivation. PLoS Pathog..

[122] Yao Y., Zhang Y., Shi M., Sun Y., Chen C., Niu M., Zhang Q., Zeng L., Yao R., Li H. (2018). Blockade of deubiquitinase USP7 overcomes bortezomib resistance by suppressing NF-kappaB signaling pathway in multiple myeloma. J. Leukoc. Biol..

[123] Hui K.F., Leung Y.Y., Yeung P.L., Middeldorp J.M., Chiang A.K. (2014). Combination of SAHA and bortezomib up-regulates CDKN2A and CDKN1A and induces apoptosis of Epstein–Barr virus-positive Wp-restricted Burkitt lymphoma and lymphoblastoid cell lines. Br. J. Haematol..

[124] Kaufman J.L., Mina R., Jakubowiak A.J., Zimmerman T.L., Wolf J.J., Lewis C., Gleason C., Sharp C., Martin T., Heffner L.T. (2019). Combining carfilzomib and panobinostat to treat relapsed/refractory multiple myeloma: Results of a Multiple Myeloma Research Consortium Phase I Study. Blood Cancer J..

[125] McConkey D.J., Zhu K. (2008). Mechanisms of proteasome inhibitor action and resistance in cancer. Drug Resist. Updat..

